# The urine examination: a modern technological test with a very ancient history

**DOI:** 10.1515/almed-2025-0121

**Published:** 2025-09-01

**Authors:** Massimo Daves, Erika Jani, Rossella Panella, Ruggero Buonocore, Stefano Pastori, Vincenzo Roccaforte

**Affiliations:** Laboratory of Clinical Biochemistry (SABES-ASDAA), Hospital of Bolzano, Bolzano, Italy; S.C. Analisi Chimico Cliniche e Microbiologiche, ASST Nord Milano, Ospedale Bassini, Cinisello Balsamo, Italy; U.O. Biochimica, Laboratory Medicine Department, Hospital Guglielmo da Saliceto of Piacenza, Piacenza, Italy

**Keywords:** urine, flow cytometry, urine test, automated urine sediment

## Abstract

For centuries, the only ‘semeiological and laboratory’ test that the physician had at his disposal to point towards to a diagnosis was the urine test. It was based on the chemical and physical properties evaluation of the sample. During the last decade of the 20th century, the use of flow cytometry opened new perspectives in the diagnostic strategy of urine examination, making it possible to automate a typically manual procedure that was represented by reading the urine sediment under an optical microscope. The combination of fully automated physical chemical and urinary sediment testing has increased the amount of information we can obtain from a simple urine test. Today’s fully automated analyzers have an accurate cell count and an excellent particle differentiation which brought a higher diagnostic reliability. Today’s uranalysis is the product of many scientists, chemists, physicians, that over decades have developed, increased the accuracy, have tried to understand, and give a meaning to all aspects (physical, chemical, and morphological) that can be found in a simple urine test. Our hope is that the urine test will be increasingly re-evaluated for the enormous diagnostic potential it can offer, also in view of a medicine that is increasingly geared to the individual needs of each patient.

In 1948 Thomas Addis, the great Scottish physician and scientist who is considered the father of modern nephrology, stated that the urine examination is the most important part of every patient’s physical examination [1]. Urine is a fluid with a highly variable chemical composition that reflects the patient’s metabolic status. This status is influenced by nutritional factors, hydration status, medications, and their metabolites. The analysis conditions are variable depending on the matrix effect. Urine is also an unstable fluid because many components are subject to significant changes. Today, the urine test is one of the most popular laboratory tests and is indicated in the suspicion or follow-up of urinary tract infections, suspicion, or follow-up of non-infectious pathologies of the urinary system, primary or secondary to systemic diseases and special cases as diabetic subjects, patients with recurrent stones, special metabolic conditions [2].

For centuries, the only ‘semeiological and laboratory’ test that the physician had at his disposal to point towards a diagnosis was the urine test. Moreover, urine has always been an easily obtainable material to be carefully studied by physicians and scientists, in the past. For this reason, it was one of the most important subjects that the physician absolutely had to dominate: uroscopy. From the Greek: okron=urine + skopéo=I observe, I analyze. Uroscopy is the urine examination that constituted the diagnostic means by excellence for centuries.

The description of the various aspects of urine: color, density, shades, transparency, odour etc. have been the subject in the past of hundreds of diagnostic treatises and medical books of the past.

## Historical milestones

**Figure 1:**
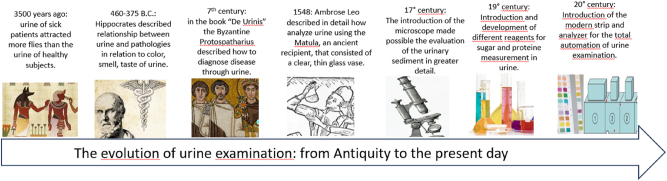
Evolution of urine test over time.

More than 3,500 years ago, the physicians of ancient Egypt and India were able to diagnose diabetes through the observation of increased urine production by the sick subjects and by the interesting observation that the urine of these patients attracted more flies than the urine of healthy subjects (Figure 1). Furthermore, the urine of these patients tasted sweet. Already several centuries before Christ, Hippocrates (460–375 B.C.) in his treatise “Prognosticon”, encourages uroscopy by emphasizing its importance in the practice of medicine. In his medical treatises, Hippocrates had already described well-defined schemes for analyzing the relationship between urine and various pathologies, especially in relation to the color, density, smell, and taste of urine. Hippocrates’ disciples believed that urine was a waste product through which the human body tried to eliminate the substance that caused the disease (Figure 1). A few centuries later, the great Greek physician Galen (129–216 A.C.), emperor Marcus Aurelius’ personal physician, tied the ureters of live animals to show how urine came from the kidneys.

But neither Hippocrates nor Galen had in fact created areal independent uroscopy doctrine.

Byzantine doctors were the first to give an autonomous dimension to this knowledge, gradually expanded the topic as the centuries passed [3].

In the 7th century, the Byzantine physician Theophilus Protospatharius (presumably in the 7th century, the certain date is not known) wrote a very popular book on uroscopy “*De Urinis”.* In his treatise the author described in detail how to diagnose a variety of diseases through urine [3] (Figure 1).

Isaac Judaeus Israeli (832?−932 A.C.) was a Jewish physician and philosopher. He was the first to describe the glomerular filtration and tubular secretion concept. He also developed a complicated flow chart that he claimed could determine every known disease [4].

Giovanni Attuario (1275–1328) was a Byzantine physician and writer who pushed observation much further than his predecessors. He wrote the best ancient work on urine, which was never written in Greek, and it was translated into Latin some two centuries later by Ambrose Leo (De urinis, Paris, 1548). He described with details how to analyze the urine in detail. The “Matula”, an ancient recipient, that consisted of a clear, thin glass vase enclosed in a straw band with a lid and handle was used to examine the urine: sediment, blood, pus, and presence of airbubbles [5], 6]. The Doctors were able to distinguish between sedimenting materials (ipostasi: from the Greek: hypo=under + stasi=to stay), surface materials (nubecolae) and suspended materials in the middle region (sublimia: from the Greek: hypsos=height and to megaloprepe=magnificence). The urine color was an essential detail. 20 colors were described in these “Matula color wheel” [7] (Figure 1).

Iconography, especially in the Middle Ages, often depicts a doctor in the act of observing against the light the urine contained in the Matula that became one of the emblems of doctors. As a curiosity, the importance that is still recognized today for this ancient instrument of the medical art is evident in the fact that the logo of *Deutsche Gesellschaft für Urologie (German Society for Urology), *of the* Società Italiana di Patologia Clinica e Medicina di Laboratorio (Italian Society of Clinical Pathology and Laboratory Medicine) *and* American Urological Association* is with the ancient physician that observe the urine in the Matula.

In 1590 Dutch lens manufacturers have produced the first optical microscope which was later improved by Galileo Galilei. Improvements made by Antonie van Leeuwenhoek (1632–1723), a Dutch optician and naturalist, allowed a deeper and better observation in the microscopic mode allowing the development of numerous new investigations into the composition of various biological liquids through microscopic observation [8].

The introduction of the microscope in the 17th century, made possible to observe the components of the urinary sediment in greater detail, leading to a better understanding of its diagnostic importance (Figure 1).

Physicians such as Marcello Malpighi and Thomas Willis were among the first to study the components of urinary sediment under the microscope and correlate them with specific diseases.

Marcello Malpighi (1628–1694) was an Italian physician and biologist. He used for almost 40 years the microscope to describe the major types of plant and animal structures and therefore marked out major areas of research in botany, embryology, human anatomy and pathology for the next generations of biologists. He also discovered the renal glomeruli, urinary tubules, and among other discoveries that the blood passes through the capillaries (plasma filtration in urine) [9].

Thomas Willis (1621–1675) was an English physician who played an important role in the history of anatomy, neurology, and psychiatry. In his book ‘Pharmaceutica Rationalis’ from 1674, Willis first described the sweet taste of the urine of diabetic patients. This led to the discovery of diabetes mellitus [10].

It is only in the late 18th and early 19th century that the sensorial evaluation of urine was replaced by a “more modern” chemical analysis. From the 1830s to 1860s occurred the transition from traditional uroscopy to modern urine examination [11] (Figure 1).

The physician Richard Bright (1789–1858) was the first to describe a specific kidney disease with the microscopic sedimentand also the variation in the laboratory signs identifying the protein in blood and urine: he described in detail the condition which is currently known as “nephrotic syndrome”, but for nearly one hundred years was known as “Bright’s disease” [12]. Interestingly, Bright’s colleagues at the time included doctors such as Thomas Addison and Thomas Hodgkin; we can say that it was certainly a very stimulating academic medical environment [12].

Golding Bird (1814–1854) was a British medical doctor. He published in “*London medical gazette”* a very comprehensive paper on components of the urinary sediment that at the time he called urinary deposits (*Lectures on the physical and pathological characteristics of urinary deposits, delivered at Guy’s hospital*) [11]. In the same years the first chemical reaction to determine glycosuria and albuminuria was developed. Fehling’s reagent was a specific reagent for determining glucose concentrations in blood and urine, developed in 1848 by German chemist Hermann von Fehling (1812–1885). For years this reagent was used to diagnose the diabetes mellitus [11].

Johann Florian Heller (1813–1871), an Austrian chemist, who is considered one of the founders of clinical chemistry, developed in 1852 the Heller’s ring test for measure albumin in the urine.

Georges Hubert Esbach (1843–1890), a Parisian doctor, facilitated the determination of albuminuria in urine by inventing a specific graduated instrument, the Esbach albuminometer and improving the reagents used in the chemical reaction required to quantify protein levels in urine [13]. The Esbach albuminometer was used in clinical chemistry laboratories until the first decades of the 20th century, described in manuals from the 1970s and reported in laboratory glassware catalogs over the years’ 80 [13].

Edme-Jules Maumené (1818–1898), a French chemist, carried on s detailed study of the physical and chemical properties of sugar from the 1850, and developed a new analytical method for the determination of glucose in different fluids, including urine, using the tin on different materials such as paper and merino wool. If sugars were present, the tissue would become brown, black, more or less intense [14].

These pioneering studies paved the way for the development of new techniques for urine analysis in the following years. The Austrian chemist Fritz Feigl (1891–1971) developed methods for spot testing on filter paper in the early 20s. It opened the way for modern urine test strips, thanks also to the development of powdered reagents for urinalysis made by the British physician William Pavy (1829–1911) and the creation of “Urinary Test Papers” for albumin and glucose by the British George Oliver (1841–1915). Frits Feigl created a new analytical chemistry area enhancing color reactions using the capillary properties of filter paper, which represents a fundamental step in the further development of the urine test [15].

The second half of the 20th century saw the birth of test strips (dipsticks). First triumphal progress began with “Clinistix” by Ames Company (today Bayer Diagnostics) in 1956. Alfred Free (1913–2000) and Helen Free (1923–2021), released in 1956, the first test specific for glucose (Figure 1).

In 1957, Ames Company introduced another dip and read test for protein in urine, Albustix. The challenge was to combine reagents for two or more tests on the same strip (one strip). In 1957, was released Uristix, which combined two tests, glucose, and protein, on one strip. By 1981 the Free couple had developed Multistix, 10 different clinical tests on a single strip. This represented a true revolution in the urinary diagnostics.

## Modern automation and clinical applications

In addition to the revolution that has taken place in the physical and chemical examination of urine thanks to the multi-parametric urine test strips, in the 1970s–1980s, the diagnostic usefulness of microscopic examination of urine sediment (hematuria and cylinders) was re-evaluated.

At the Joint Meeting of the American Society of Clinical Pathologists, held in Las Vegas in 1977, the long debate began on whether it should be required to include microscopic examination in routine urine tests [11].

The National Committee for Clinical Laboratory Standards (NCCLS) has been trying to standardize urine examination by producing guidelines since 1985 with the aim of standardizing and improving urinary examination procedures [16].

The Italian pathologist Angelo Burlina in 1992 defined the standard urine examination as ‘a truly organ-metabolic profile, which explores not only the morpho-functional state of one organ, the kidney, but also many metabolisms, from hydro-electrolytic to glucidic to protidic, etc.’ He considered the physical-chemical examination as a basic urinary profile and the microscopic examination as an in-depth examination [17].

During the last decade of the 20th century, the use of flow cytometry opened new perspectives in the diagnostic strategy of urine examination making it possible to automate a typically manual procedure that was represented by reading the urine sediment under an optical microscope [11]. Over the last years, increasingly high-performance automated urine sediment readers based on the automatic reading of microscopic images or by cytofluorimetry have appeared on the market. In the latest generation of analytical systems, the two different technologies of automated readers can even be sequenced into integrated analytical systems, consisting of automated readers of the chemical/physical examination and urine sediment.

Automation of the urinary examination has significantly reduced human laborand allowed for greater standardization of results. A more accurate particle counts in the urine allowed a better differential diagnosis between patients with kidney or urinary tract diseases and healthy patients [18].

In clinical practice, urine examination is required in cases of suspected urinary infection, screening or follow-up of kidney disease, non-infectious urinary tract diseases (primary or secondary systemic diseases, including rheumatic diseases, hypertension, pregnancy, or side effects of drugs) and in cases of recurrent kidney stones. Urine examination is also frequently required in emergency situations to assess dehydration or volemia (urine concentration), in suspected cases of urinary infection (leucocyte esterase and the presence of nitrites), in cases of diabetic ketoacidosis (glucose concentration and ketones), for the assessment of urinary tract bleeding (hematuria). [19]. The combination of fully automated physical chemical and urinary sediment testing has increased the amount of clinical information we can obtain from a simple urine test. Today’s fully automated analyzers provide an accurate cell count and an excellent particle differentiation which brought a higher diagnostic reliability.

The urine sediment examination requires an appropriate approach based on correct methodology, appropriate equipment, knowledge, experience, and up-to-date skills [20]. The latest automated urine sediment analyzers combine impedance measurement technology with cytofluorimetry. The sample passes through two flow cells, one dedicated to the analysis of microorganisms and the other dedicated to the analysis of all other corpuscular elements. In addition, to characterize a sample, the conductivity of the solution is measured, which is an indication of the concentration of electrolytes in the urine [21].

The last international guidelines seek to raise awareness of the importance of professional expertise in the field of urinary morphology and the importance of interaction with clinicians; to implement a program for the assessment of morphological competence, to stimulate research and development of instrumental methodologies suitable for the needs of clinical diagnosis [22].

Over time, guidelines have attempted to standardize the method of analysis, time and manner of collection, patient preparation, interferences that could affect the result, contamination, transport, storage, diagnostic strategies related to the presence of various particles in urine. The latest European urinalysis guideline is in this respect a fundamental reference for all stakeholders interested in urine testing [22]. A fundamental aspect that specialists in laboratory medicine must be very familiar with is the pre-analytical phase of the urine test. The European Urinalysis Guidelines describe in detail the different aspect that may affect the quality of the result of urinalysis, as specimen collection and preservation, stability, transport, collection containers, handling of the specimens and it is imperative that laboratory medicine governs all these fundamental aspects in order to obtain accurate analytical results useful for proper patient management [23], 24].

Obviously, another aspect that the laboratory medicine specialist must be familiar with is the analytical aspect, the diagnostic significance of the test strips and their limitations and the possibility of false positive or false negative results. The same applies to urine sediment examination with the new automatic instruments, and the need to maintain a morphological competence in the optical microscopic recognition of the various components of the urinary sediment [22], 25], 26].

Last but not least the Italian Interdisciplinary Urinalysis Group underlined the importance of the postanalytical phase in physical, chemical and morphological urine examination. This group provide some recommendations to improve and standardize the laboratory report on the urine examination, with the indication of analytical methods, of units of measurement and reference values, as well as to improve the interpretation of dip stick urinalysis with particular regard to the reconsideration of the diagnostic significance of the evaluated parameters together with an increasing awareness of the limits of sensitivity and specificity and the possible interference of this analytical method [27].

The physical-chemical and morphological examination of urine is a widely used test because of the wealth of information it can provide, the ease with which the sample can be collected, the possibility of performing it in any laboratory in a practical, accurate and safe manner, and its cost-effectiveness [21].

The diagnostic strategies currently practiced are the result of the history of the technological age of urine examination [11].

In recent years, new automated analyzers have tended to increase the accuracy of the urinary examination and offer more and more information that could help the clinician in his or her diagnosis.

Early detection of albumin (and/or proteinuria) and albuminuria/creatinuria with proteinuria/creatinuria ratios, with sensitive and accurate methods, have provided additional help for early diagnosis of kidney disease and cardiovascular prevention [21]. Since 2002 the Clinical Practice Guideline for the Evaluation and Management of Chronic Kidney Disease (CKD) serves to update the evaluation, management, and treatment of all patients with CKD. In the Kidney Disease: Improving Global Outcomes (KDIGO) guideline, the importance of measuring urine albumin as an essential marker in the management of the patient with nephropathy is emphasized for the first time [28].

In the light of the knowledge accumulated over the last few decades, we can now state that many parameters usually measured in urine are either of no clinical use or are only useful in certain conditions (parameters useful clinical conditions: glucose, ketones; parameters not useful: bilirubin, urobilinogen) [21].

Recently, the new generation of automated urine particle analyzer has been widely evaluated. The conclusions of the various studies are that these analyzers are very accurate in detecting urine particles related to pathological processes with high analytical performance [29], 30].

Automatic analyzers for the evaluation of the corpuscle part have both some advantages and some disadvantages. The quantification of corpuscular elements performed with automatic analyzers is more repeatable than one performed by a human observer under a microscope [29], 30].

The possibility of quantifying the elements per unit of volume examined makes it possible to standardize the analysis and provides an objective indicator for clinical evaluation [21].

The introduction of automatic analyzers to count the corpuscle fraction has, paradoxically, increased the need for operators’ morphological expertise. In fact, automated urine sediment analyzers are not able to recognize sufficiently accurately some important components of the urine sediment such as lipids, protozoa, crystals, casts giving generic alarms that need to be revised [21], [31], [32], [33]. In addition, the laboratory medicine specialist must be able to correctly identify the different types of crystals, including rare and atypical ones, by eventually adopting the necessary procedures [32].

The introduction of the new parameter (Urinary Red Blood Cell distribution) that measures erythrocyte size, with the third generation of automated analyzers, allows a rapid, objective differentiation between glomerular and non-glomerular hematuria in combination with clinical data [34]. As has always been the case, microscopic examination remains the gold standard for the determination of dysmorphic erythrocytes in urine. Recently, Wang et al. reported that the identification of atypical cells in urine sediment by an automated urine sediment analyzer may be useful for the detection of bladder cancer [35].

With the evolution of urine examination methods, the automation of the analysis of the corpuscular part, and the increased analytical accuracy, this examination has begun to regain the respect it was given millennia ago. It is important to emphasize that the improvement in analytical accuracy and reproducibility may be adversely affect if the pre-analytical phase is not well controlled [22], 24], 25]. The importance of proper management of the pre-analytical phase of the urine examination was emphasized by Clinical and Laboratory Standard Institute as early as 2009: the CLSI GP16-A3 document is written for laboratory and non-laboratory personnel which are responsible for the collection, transport, and analysis of urine specimens [36]. Clinical laboratories must check that pre-analytical quality specifications are met to ensure reliable results. In this regard, authors have recently proposed specific cutoffs for epithelial cell and bacterial counts performed with automated urine sediment analyzers that can be used as quality indicators of proper urine collection [37].

Today’s urine exam is the product of many scientists, chemists, physicians, that over decades have developed, increased the accuracy, have tried to understand, and give a meaning to all aspects (physical, chemical, and morphological) that can be found in a simple urine test. Our hope is that the urine test will be increasingly re-evaluated for the enormous diagnostic potential it can offer, also in view of a medicine that is increasingly geared to the individual needs of each patient.
